# Isolation of Lactic Acid Bacteria (LAB) from Salmonids for Potential Use as Probiotics: *In Vitro* Assays and Toxicity Assessment of *Salmo trutta* Embryonated Eggs

**DOI:** 10.3390/ani14020200

**Published:** 2024-01-07

**Authors:** Augusto Vargas-González, Miguel Barajas, Tania Pérez-Sánchez

**Affiliations:** 1 Biochemistry Area, Health Science Department, Faculty of Health Sciences, Public University of Navarra, 31008 Pamplona, Spain; miguel.barajas@unavarra.es; 2 Official College of Veterinary Surgeons of Huesca, 22004 Huesca, Spain; taniapersan@gmail.com

**Keywords:** probiotics, lactic acid bacteria, salmonids, *Salmo trutta*, embryonated eggs, fish pathogens, antimicrobial resistance, antagonistic assays

## Abstract

**Simple Summary:**

Due to the increase in antibiotic resistance observed in pathogenic bacteria, new alternatives are being explored to reduce the use of these drugs. Probiotics, a group of bacteria that includes lactic acid bacteria (LAB), are capable of stimulating and strengthening the immune system and have showed antagonistic effects against various pathogenic bacteria. The aim of this study was to identify and test potentially probiotic LAB, isolated from Brown trout and Rainbow trout, to select those with the least antibiotic resistance, the best antagonistic effect against fish pathogens, and minimal negative effects during their application on embryonated Brown trout eggs. As a result, various bacterial strains were obtained that proved to be safe in terms of antibiotic resistance and effective against several of the pathogens to which they were exposed. Nevertheless, only a limited number of these strains were found to be safe for use in Brown trout eggs. However, some findings suggest the need to investigate the possibility that certain bacterial strains considered probiotics might have adverse effects on fish health.

**Abstract:**

This research investigates the potential of lactic acid bacteria (LAB) from freshwater salmonids as prospective probiotics for application in aquaculture. LAB and pathogenic bacteria were obtained from mucus and tissues of *Oncorhynchus mykiss* and *Salmo trutta* from fish farms in northeast Spain that had not used antibiotics for the six months preceding the study. Isolates were identified using Gram staining and sequencing of 16S rRNA and ITS-1. To assess the safety of the LAB, antibiotic susceptibility tests (ASTs) against 23 antimicrobials were performed. *In vitro* antagonism assays were conducted to evaluate the inhibitory effects of living LAB using the agar diffusion test method and their metabolites using the agar well diffusion method. The assays targeted six specific pathogens: *Aeromonas salmonicida* subsp. *salmonicida, Carnobacterium maltaromaticum, Vagococcus salmoninarum, Yersinia ruckeri, Lactococcus garvieae*, and the marine pathogen *Vibrio jasicida.* Additionally, a toxicity assay was conducted on embryonic eggs of *S. trutta*. The ASTs on probiotic LAB candidates revealed varied responses to antimicrobials, but no resistance to oxytetracycline or florfenicol, which are two antibiotics commonly used in aquaculture, was detected. The *in vitro* assays indicate that LAB exhibit antagonistic effects against pathogens, primarily when directly stimulated by their presence. In applications involving embryonic eggs or larvae, certain live strains of LAB were found to have adverse effects, with some isolates resulting in higher mortality rates compared to the control group or other isolates. Furthermore, the potential pathogenicity of certain LAB strains, typically considered safe in salmonids, warrants deeper investigation.

## 1. Introduction

The most well-known genera associated with salmonids are *Oncorhynchus* and *Salmo*. The main commercial species are *Salmo salar* (Atlantic salmon), *Oncorhynchus mykiss* (Rainbow trout), and *Oncorhynchus kisutch* (Coho salmon). On the other hand, *Salmo trutta* (Brown trout) is usually bred for sport fishing and conservation purposes. The main global producers of salmonids are Norway and Chile, followed by Canada, the UK, and the Faroe Islands. The global production of *O. mykiss* in 2022 was 952,691 metric tons, and in Spain, the production of *O. mykiss* reached 16,328 metric tons, valued at EUR 43.6 million, mainly in freshwater farms. Regarding the production of *S. trutta,* its breeding is commonly dedicated to river and lake restocking programs [1].

Due to the economic importance of these species, numerous studies have been conducted to better understand the pathogens that affect them and the ways to treat them. The main pathogens of interest in freshwater aquaculture are *Carnobacterium maltaromaticum*, *Vagococcus salmoninarum*, *Lactococcus garvieae*, *Flavobacterium psychrophilum*, *Aeromonas salmonicida* subsp. *salmonicida*, and *Yersinia ruckeri* [2,3,4,5,6,7,8,9,10,11].

In farms experiencing outbreaks of these bacterial diseases, some of the most commonly antibiotics used in freshwater aquaculture include oxytetracycline, florfenicol, and flumequine. These antibiotics can be administered orally or by direct intramuscular injection, the latter being mainly performed in breeders. However, the need to seek new alternatives to the use of antibiotics, due to the emergence of bacteria that are resistant or multi-resistant to these drugs, has led to research projects being conducted in order to obtain biological control alternatives [12]. These studies range from synthesizing new molecules with antibacterial properties to employing extracts and compounds derived from plants, utilizing bacteriophages, or using probiotic bacteria [13].

Lactic acid bacteria (LAB) are a group comprising different species of Gram-positive bacteria that have been isolated from various environments and are very important in the food industry. Many research efforts have been made to isolate and identify LAB probiotics from salmonids or to test the effects of these on this species [14,15]. In this sense, one of the most studied is *Pediococcus acidilactici* MA18/5M (Bactocell^®^), which is isolated from pasture gramineae and is the first and, to date, only lactic acid and probiotic bacteria authorized by the European Union’s Standing Committee on the Food Chain and Animal Health for use as a zootechnical feed additive in salmonids and shrimps [16].

The European Food Safety Authority (EFSA) has established criteria for the use of probiotic bacteria as additives in animal feed. Many LAB are classified within the group known as Qualified Presumption of Safety (QPS), a generic safety pre-assessment approach that covers safety concerns for humans, animals, and the environment, and which facilitates the initiation of authorization processes [17,18]. Then, to start the authorization process, the reporting of four mandatory scientific criteria is required: identification of the microorganism, antimicrobial susceptibility, antimicrobial production, and toxigenicity and pathogenicity [19]. The identification “can be achieved by comparing the sequences commonly used for taxonomic identification (e.g., 16S rRNA gene)”. For the antimicrobial susceptibility, “a phenotypic testing is required based on determination of a minimum inhibitory concentration (MIC)” for ampicillin, vancomycin, gentamycin, kanamycin, streptomycin, erythromycin, clindamycin, tetracycline, and chloramphenicol, antimicrobials considered relevant to their use in humans and animals. The “assessment of the antimicrobial properties of the bacteria” is not required if it is classified as QPS, “a series of tests should be made to assess the inhibitory activity of culture supernatants against reference strains known to be susceptible to a range of antibiotics and if there is a positive outcome in one or more species, the inhibitory substance should be identified”. To evaluate toxicity, “the information relating to toxigenicity should be provided for active agents and production strains, including history of use of the strain or any close relative”, and when the additive is intended to be used in salmonids, it is permitted to use salmon and trout as animal models [20,21].

Embryonated eggs from *O. mykiss* are used as a standard when studying the toxicological effects of different substances in freshwater aquatic environments to evaluate the chronic and acute toxicity of these substances and as a model for assessing toxic effects in the early life stages of salmonid fish [22]. However, *S. trutta* is more sensitive to some pollutants than *O. mykiss* and serves as an excellent indicator of environmental ecosystem health [23]. Depending on the genetic line, the hatching of *S. trutta* takes, from fertilization, up to 406 degree-days (if consistently maintained at 10 degrees Celsius, it would equal approximately 41 days until hatching). Once hatched, the vesicular larvae may require up to 610 degree-days from fertilization to reach the stage of free-swimming and self-feeding [24].

The aim of this work was to explore the potential of using the endogenous LAB from the microbiota of freshwater salmonids as probiotics and alternatives to the use of antibiotics against six key pathogens in aquaculture. This included five significant freshwater bacterial pathogens—*C. maltaromaticum*, *V. salmoninarum*, *Lactococcus garvieae*, *A. salmonicida* subsp. *salmonicida*, *Y. ruckeri*—as well as the marine bacterium *Vibrio jasicida*. We tested their antibiotic susceptibility and antagonistic effects *in vitro*. Additionally, the toxicity of these isolates was assessed *in vivo* using embryonated *S. trutta* eggs. These eggs were incubated in cell culture flasks, a method inspired by previous research that successfully employed fish larvae for microbiota studies [25,26,27,28,29]. This approach allowed us to closely monitor and assess the effects of the LAB strains, providing a controlled examination of their potential and their impact on the early developmental stages of *S. trutta.* The methodology aligned with the four requirements of the EFSA—identification of the microorganism, antimicrobial susceptibility, antimicrobial production, and toxigenicity—for the authorization of a probiotic as a commercial additive.

## 2. Materials and Methods

### 2.1. Isolation of LAB and Pathogens from Fish

**Figure 1:**
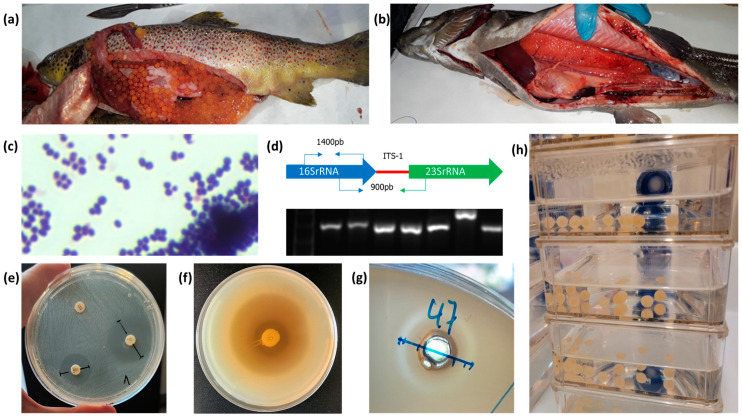
(**a**) *S. trutta*, (**b**) *O. mykiss*, (**c**) Gram stain, (**d**) 16S ribosomal RNA gene and ITS-1 PCR amplification, (**e**) AST, (**f**) antagonistic *in vitro* assay agar plug diffusion method, (**g**) antagonistic *in vitro* assay agar well diffusion method, (**h**) toxicity assay on *S. trutta* embryonated eggs.

Fish farms in the northeast of Spain, dedicated to the commercial breeding of *O. mykiss* or restocking of *S. trutta*, were visited on routine veterinary controls. Among these farms, particularly those that refrained from antibiotic use in the six months preceding the veterinary controls, fish undergoing routine sanitary checks were utilized to obtain isolates of probiotic bacteria for our research. Samples were taken from animals of different age groups, ranging from 20 g juveniles to 3 kg breeders, in order to obtain greater diversity in the isolates. In cases where a bacterial infection was suspected, samples were also collected from the diseased fish to obtain pathogenic bacteria for further antagonistic assays against the probiotic bacteria (see Figure 1).

In order to obtain the LAB, sterile swabs or razor blades were used to collect samples of mucus from gills, mouth, the distal portion of intestine, or skin from healthy fish [14]. The swabs were incubated in MRS broth (de Man Rogosa, Sharpe broth, Scharlab, Spain). Next, to obtain isolated colonies, loopfuls of each bacterial culture were streaked onto MRS agar plates using the streak plate technique. These colonies were selected and picked with a sterile loop and sub-cultured in MRS broth. After incubation, a loopful of each culture was used for microscopic identification of Gram-positive organisms (see Table 1).

When a moribund fish was detected with evident external lesions, such as exophthalmia, boils, erosions, petechiae, etc., tissue samples were taken from liver, spleen, head kidney, swim bladder, or skin lesions such as wounds, pustules, or boils [30]. To obtain bacteria suspected of being pathogenic, the samples were inoculated into TSB (tryptone soy broth, Scharlab, Spain), AOE broth (Anacker and Ordal’s enriched broth), or when a Gram-positive pathogen was suspected, in MRS broth, and they were incubated at room temperature. Next, to obtain isolated colonies, loopfuls of each bacterial culture were streaked onto agar plates using the streak plate technique and incubated. The colonies were picked with a sterile loop and sub-cultured in TSB, AOE broth, or MRS broth. After incubation, a loopful of each culture was used for Gram staining and microscopic observation of the isolated bacteria (see Table 1). When there was the suspicion of a pathogen that requires lower temperatures, the incubation procedure was conducted at 15 °C.

Bacterial pellets were obtained by centrifugation of the liquid cultures and subsequently resuspended in TS or MRS broth supplemented with 15% *w/v* glycerol. The suspension was then promptly stored at −80 °C to ensure optimal preservation of the bacterial stock cultures.

### 2.2. Genomic Bacterial DNA Extraction

Genomic bacterial DNA was extracted from 2 mL of 24 h pure cultures using a QIAamp DNA Mini Kit™ (QIAGEN) following the manufacturer’s instructions. For Gram-negative bacteria, a prior digestion step was performed using 180 µL of 20 mg/mL lysozyme (dissolved in Tris-HCl buffer, pH 8.3, 2 mM EDTA, and 1.2% Triton), incubated at 37 °C for 1.5 h with agitation at 1000 rpm, while for Gram-positive bacteria, the incubation was extended to 3 h. The DNA elution was performed with nucleases-free water and quantified using a NanoDrop One™ device (Thermo Fisher Scientific, Waltham, MA, USA). The obtained DNA was temporarily stored at −20 °C.

### 2.3. PCR Identification of Presumptive Fish Pathogenic Bacteria from the Isolates

For the initial identification of fish pathogenic bacterial isolates, TaqMan™ probe-based qPCR tests were performed on an CFX Connect Real-Time PCR System (Bio-Rad Hercules, CA, USA) with iTaq Universal Probes Supermix 2X (Bio-Rad) for the detection of *Y. ruckeri*, *F. psychrophilum*, *Lactococcus garvieae,* and *Renibacterium salmoninarum* [4,8,10,31,32]. The iTaq Universal SYBR Green Supermix (Bio-Rad) was used for the detection of *A. salmonicida* subs. *salmonicida*, *C. maltaromaticum,* and *V. salmoninarum* [3,5,9,33]. Conventional PCR was performed on an PTC-200 Peltier Thermal Cycler (MJ Research) with 2X KAPA2G Fast Hotstart ReadyMix (Kapa Biosystems) and agarose gel electrophoresis for the detection of *Flavobacterium columnare* [11]. As an endogenous control, the primers and probe for ELF-1α described by Sepulveda et al., 2013, were used [34]. The primers and probes are described in Table 2.

### 2.4. Molecular Identification of the Potential LAB Probiotic Isolates and of Presumptive Fish Pathogenic Bacteria by Partial Sequencing of the 16S rRNA gene and ITS-1 Intergenic Spacer

Bacterial 16S rRNA gene was amplified by conventional PCR performed on an PTC-200 Peltier Thermal Cycler (MJ Research) using 2X KAPA2G Fast Hotstart ReadyMix (Kapa Biosystems), and the standard 16S ribosomal RNA gene primers 27F (5’-AGA GTT TGA TCC TGG CTC AG-3’) and 1492R (5’-ACG GCT ACC TTG TTA CGA CTT-3’) were used [35]. The intergenic spacer ITS-1 (16S rRNA-ITS-23S rRNA) was amplified using the primer set R1391 (5’-TTG TAC ACA CCG CCC GTC-3’) and the reverse complementary sequence of primer 473F described by Gürtler et al., 2013, (5’-CTT TCC CTC ACG GTA CT-3’) [30]. The PCR reaction for the ITS-1 rRNA was performed using 12.5 µL of 2X KAPA2G Fast Hotstart ReadyMix (Kapa Biosystems), 1.5 µL of each primer at a concentration of 10 µM, 7 µL of nuclease-free water, and 3 µL of template (approximately 100 ng). The thermal cycling conditions were as follows: initial denaturation at 95 °C for 3 min, followed by 35 cycles of denaturation at 95 °C for 15 s, annealing at 55 °C for 15 s, and extension at 72 °C for 2 min, followed by a final extension at 72 °C for 2 min. The PCR products were analyzed by agarose gel electrophoresis, and the observed bands of expected size were purified using a NucleoSpin Gel and PCR Clean-Up Kit (Macherey-Nagel, Germany). The purified PCR products were sent to Macrogen, Spain (Madrid), for sequencing.

The received sequences were subjected to BLAST analysis on the NCBI (National Center for Biotechnology Information) website and further compared using the web service available at EzBioCloud (https://www.ezbiocloud.net/identify accessed on 14 November 2023) [36]. The Molecular Evolutionary Genetics Analysis (MEGA) software, version 7.0.26, was utilized to validate the base calling process and subsequently perform the assembly of forward and reverse sequences, thereby generating extended sequences. The assembly was specifically conducted to bridge the overlapping regions between the 16S ribosomal RNA gene, intergenic spacer, and a portion of the 23S ribosomal RNA gene.

Phylogenetic analysis was also conducted using MEGA 7.0.26 against the sequences displayed in Table 3, first by alignment of the sequences using the MUSCLE tool (Multiple Sequence Comparison by Log-Expectation) and adjusting to the shorter sequence and then using the maximum likelihood method with a bootstrap test of 10.000 replicates [37,38,39].

### 2.5. Identification of Presumptive Fish Pathogenic Bacteria from the Isolates by MALDI-TOF

In addition to the identification of isolates by PCR and sequencing of 16S and ITS-1, some of the presumptive fish pathogenic bacteria isolates (*Y. ruckeri*, *C. maltaromaticum*, *A. salmonicida* subsp. *salmonicida*, *Lactococcus garvieae*, *V. salmoninarum*) were sent to be identified by MALDI-TOF MS (Bruker Daltonic GmbH, Bremen, Germany). The analysis was conducted at the University of La Rioja, Spain.

### 2.6. Antibiotic Susceptibility Test Using Disks on LAB Probiotic Candidates

The LAB isolates probiotic candidates were subjected to a test to determine their susceptibility patterns to antibiotics using the agar diffusion method. Twenty-three different antimicrobial susceptibility test discs (Oxoid, UK) were used: vancomycin (30 µg), ciprofloxacin (5 µg), amoxicillin/clavulanic acid (30 µg), kanamycin (30 µg), nalidixic acid (30 µg), clindamycin (2 µg), doxycycline (30 µg), erythromycin (15 µg), oxacillin (1 µg), cefuroxime sodium (30 µg), florfenicol (30 µg), nitrofurantoin (300 µg), ceftriaxone (30 µg), neomycin (30 µg), tetracycline (30 µg), flumequine (30 µg), penicillin G (10 units), ampicillin (10 µg), streptomycin (10 µg), trimethoprim (5 µg), chloramphenicol (30 µg), gentamicin (10 µg), and oxytetracycline (30 µg). The bacteria were grown as indicated in Table 1. They were then centrifuged at 2500 rpm for 15 min, the culture medium was discarded, and they were resuspended in a PBS solution to a concentration equivalent to 1 × 10^−8^ CFU/mL, corresponding to 0.5 McFarland. Subsequently, they were seeded on TSA plates and allowed to air-dry. Antibiotic disks were placed on these plates, which were then incubated for 48 h. After incubation, the diameters of the inhibition zones created by the disks were recorded. [40,41,42].

### 2.7. Antagonistic In Vitro Assay by Agar Plug Diffusion Method of LAB

The antibacterial activity of the live LAB isolates, identified as probiotic candidates, was tested against six pathogens. *A. salmonicida* subsp. *salmonicida* and *C. maltaromaticum* were obtained from *S. trutta* in this study. The remaining pathogens, i.e., *V. salmoninarum*, *Y. ruckeri*, *Lactococcus garvieae* from *O. mykiss*, and *Vibrio jasicida* from *Dicentrarchus labrax*, were provided by the veterinarian from previous field work.

A modified version of the agar diffusion test method described by Elleuch et al. (2010) was used [43,44]. To neutralize the inhibitory effect of the lactic acid produced by the LAB, mini MRS agar plates buffered with a phosphate buffer were prepared as follows: 66 g of MRS agar (Scharlab, Spain) as per the manufacturer’s instructions, 10 g of disodium hydrogen phosphate (Na_2_HPO_4_) (Merck, Germany), and 4.3 g of monosodium dihydrogen phosphate (NaH_2_PO_4_) (Merck, Germany) were weighed using an analytical balance and dissolved in one liter of distilled water using a magnetic stirrer with a heater. Once the mixture was dissolved, 500 mL of the medium was transferred to two 1L bottles and autoclaved at 121 °C for 15 min. Subsequently, 5 mL of the medium was plated onto each miniplate, air-dried, and made ready for use. The probiotic candidates were grown as pure cultures (see Table 1), and then a 5 μL drop at a concentration of 1 × 10^8^ CFU mL^−1^ was inoculated onto the miniplates and then allowed to grow. When the LAB drops were ready, the agar with the drop was removed from the miniplates near a Bunsen burner, placing this agar in the center of standard bacteriological Petri dishes with the probiotic bacteria drop facing downwards. Fresh cultures of each pathogen were mixed with 30 mL of TSA (marine broth from Sigma-Aldrich, US, for *V. jasicida*) and poured into each standard bacteriological Petri dish for mass inoculation of the pathogens at a concentration of 1 × 10^7^ CFU mL^−1^. The plates were allowed to solidify and were then incubated. Antagonism was determined as the diameter of the inhibition growth zone.

### 2.8. Antagonistic In Vitro Assay by Agar Well Diffusion Method with Supernatants from Pure LAB Cultures

To assess the antibacterial activity of the metabolites present in the supernatant of the LAB cultures against the pathogens, the agar well diffusion method was employed [44]. The LAB and the pathogens were grown as indicated in Table 1. A 10 mL aliquot of each pure culture of LAB was centrifuged at 1500 rpm for 15 min. The supernatant was collected, neutralized to pH 7.0 using 0.1 M NaOH to eliminate the inhibitory effect of acidic pH from the lactic acid, and filtered using sterile 0.22 μm Millex™ syringe filters (Merck Millipore). It was then stored at −20 °C for subsequent use.

Fresh cultures of each pathogen (see Table 1) were mixed with 100 mL of TSA (or marine agar for *V. jasicida*) and poured into 150mm × 25mm bacteriological Petri dishes for mass inoculation at a concentration of 1 × 10^7^ CFU ml^−1^. The plates were then allowed to solidify. Using the blunt end of a sterile 1000 μL micropipette tip, wells were created in the agar. The bottoms of these wells were sealed with 10 μL of agar to prevent leakage of the supernatants being tested. Subsequently, 50 μL of supernatant was added to each well. The plates were first incubated at 4 °C for one hour to allow diffusion of the supernatants. They were then incubated under appropriate conditions, and the radii of the inhibition zones were measured from the edge of the well to the edge of the pathogen growth zone.

### 2.9. Toxicity Assay of Live Bacteria on Embryonic Eggs of Salmo trutta

Fertilized *S. trutta* eggs were procured from a restocking farm in northeast Spain. This batch of eggs consisted of a mix from 6 females and semen from 2 males, which is the usual practice at the farm. While this approach introduced genetic variability into the study, it is also representative of natural conditions. When the eggs had approximately 95 degree-days (dd) post fertilization, they were transported in polystyrene trays with ice to maintain a temperature of around 4 °C. Upon arrival at the laboratory, they were transferred to two 5 L plastic tanks containing water at a concentration of 150 ppm and a temperature of 5 °C. This water, a blend of commercial still water and deionized water, was consistent across all experiments and was regularly refreshed with 90% renewal every two days. The tanks were stored in a low-temperature incubator, maintaining this temperature until the eggs reached 280 dd. Then, the water temperature was gradually increased, raising by 1 °C per hour through successive water exchanges until each tank reached 8 °C. At 296 dd, 75 cell culture flasks, each with a vented cap, 250 mL capacity, and a surface area of 75 cm^2^, were set up. Each flask contained 100 mL of water at 8 °C and 10 eggs: 70 flasks were allocated for the trial with an additional 5 as reserves. From this point until the conclusion of the experiment, 90% of the water in the flasks was replaced with fresh water every two days, the flasks were maintained in a refrigerated incubator at 8 °C and the eggs were observed daily using an inverted microscope.

When the eggs reached 320 degree-days, the 70 flasks of 10 eggs each were randomly divided into 7 groups of 10 flasks each: 1 independent control group and 6 groups to be treated with each of the 6 LAB probiotic candidates. This setup resulted in a total of 100 eggs per condition, equating to 100 biological replicates, and with each flask serving as a technical replicate, there were 10 technical replicates per condition. The selection of these probiotic candidates was based on their species, source of isolation, antibiotic resistance profile, and their antagonistic effect against pathogens. The primary objective of this experiment was to evaluate the feasibility of administering live LAB probiotics through direct immersion on embryonated salmonid eggs.

Before each immersion bath in the experiment, overnight cultures of each LAB strain were prepared, once for each of the three baths. Each strain was revived from cryovials by inoculating a cryobead into 10 mL of MRS broth. These cultures were incubated at 25 °C for 24 h without agitation. Subsequently, the culture was mixed by vortexing, and 2.5 mL of this culture was transferred to 250 mL of MRS broth and incubated for 48 h at 25 °C with agitation. Post incubation, the cultures were centrifuged at 6000 rpm for 10 min at 18 °C. The supernatant was discarded, and the pellet was resuspended in 25 mL of phosphate-buffered saline (PBS). After vortex mixing and another round of centrifugation, the supernatant was again discarded, and the pellet was resuspended in another 25 mL of PBS. This suspension was transferred to a 50 mL centrifuge tube for further centrifugation, after which the supernatant was removed, and the pellet was reconstituted in 12 mL of PBS, preparing the LAB for use in the *S. trutta* eggs assay.

Serial tenfold dilutions were prepared from these bacterial suspensions, which were then plated on MRS agar plates, and colony counting was conducted. This confirmed that the bacterial suspensions prepared on each occasion were at a concentration ranging from 3.95 × 10^10^ to 5.65 × 10^10^ CFU mL^−1^.

In each flask, 1 mL of the LAB suspension was added to 99 mL of water and gently mixed by pipetting up and down to expose the embryonated eggs to a concentration of around 3,95 × 10^8^ to 5.65 × 10^8^ CFU ml^−1^ of LAB. For the negative control, 10 flasks with *S. trutta* eggs were treated with 1 mL of PBS in 99 mL of water. All flasks were stored in the incubator at 8 °C.

After 24 h of exposure, 95 mL of water was carefully removed from each flask. The water from every group was then combined into a single jar, one for each group. This pooling of water facilitated the measurement of several key water quality parameters, given the limitations of the available measuring equipment and the volume requirements for accurate readings. The following parameters were measured in the combined water sample: dissolved oxygen as a percentage (%); dissolved oxygen in mg/mL; pH level; conductivity in mS/cm; and turbidity in nephelometric turbidity units (NTU). The eggs were carefully rinsed three times with fresh water at 8 °C and replenished with fresh water at the same temperature.

**Figure 2:**
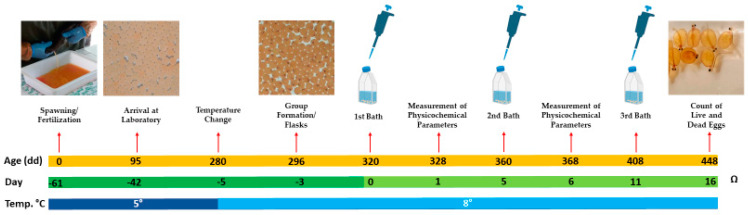
Timeline of the trial assessing the effect of LAB on *S. trutta* eggs. The illustration features three parallel lines: an orange line representing age in degree-days, a green timeline marking the days from the start of the experiment (day 0) to day 16, and backwards to −61 days, beginning with egg fertilization. The third line, in blue, is divided into dark blue for periods when the eggs were maintained at 5 °C and light blue for when the temperature was kept at 8 °C.

This procedure was repeated three times: the initial immersion occurred at 320 degree-days, the second at 360 degree-days, and the final at 408 degree-days. Unlike the first two immersions, after the 24 h of exposure following the final immersion, the physicochemical parameters were not measured. Daily observations of the eggs were conducted using an inverted microscope until day 16 of the experiment at 448 degree-days post fertilization, when the count of the number of dead and live eggs, as well as hatched eggs with live larvae, was performed, marking the conclusion of the experiment (see Figure 2).

### 2.10. Statistical Analysis

The data obtained from the toxicity assay conducted on *S. trutta* eggs was analyzed using IBM SPSS Statistics for Windows, Version 29.0.1.0. Initially, the Shapiro–Wilk test was conducted for each group of replicates treated with LAB to assess the normality of the data. As the test indicated non-normal distribution for at least one group, the Kruskal–Wallis nonparametric test was employed for comparisons among the different treated groups instead of ANOVA, which is typically used for normally distributed data. This approach was chosen to ensure the appropriate statistical aligning with the data characteristics. All statistical analyses were performed with a significance level of 95%.

### 2.11. Ethics

The developmental stages of *S. trutta* used in these experiments fell outside the scope of the European Union Directive 2010/63/EU on the protection of animals used for scientific purposes, as well as the Spanish Royal Decree 53/2013; hence, there was no need for approval from the Navarra Government’s Department for Rural Development and Environment, which oversees animal welfare and experimentation in the region.

## 3. Results

### 3.1. Isolation and Molecular Identificacion of Fish Pathogens and LAB Probiotic Candidates

Through the molecular identification of the isolates (Table 4), 17 LAB strains were identified as potential probiotic candidates: 4 strains of *P. acidilactici*, 1 of *Leuconostoc mesenteroides*, 2 of *Lactococcus lactis*, and 10 *Lactiplantibacillus* sp. Strains, showing high similarity to both *Lactiplantibacillus plantarum* and *pentosus.* Additionally, 1 isolate of *Carnobacterium divergens*, 11 of *C. maltaromaticum*, and 1 *Aerococcus* sp., exhibiting high similarity to both *Aerococcus viridans* and *urinaeequi,* were obtained from healthy animals. However, they were excluded from further assays, as they belong to genera and species associated with diseases.

When searching for pathogens, we obtained two strains of *C. maltaromaticum* (St-PS-HK-63 and St-PS-HK-64) from the head kidney of *S. trutta* displaying clinical signs of disease, and from one specimen of *O. mykiss*, a *Lactiplantibacillus* sp. from the head kidney and two isolates of *Leuconostoc mesenteroides* from the swim bladder were obtained. This *O. mykiss* specimen exhibited splenomegaly, swim bladder thickening, and liver adhesions to the muscle tissue. One strain of *A. salmonicida* subsp. *salmonicida*, labeled as St-Mu-SK, was acquired from skin boils on *S. trutta* and identified through MALDI-TOF MS, a reliable technique based on the analysis of unique protein mass fingerprints. However, at the time of writing this manuscript, confirmation through sequencing of the 16S rRNA and ITS-1 genes had not yet been completed. Additionally, four pathogens provided by the veterinarian, i.e., *V. salmoninarum* strain Om-V-L-69, *Lactococcus garvieae* strain Om-Pe-HK-61, *Y. ruckeri* strain Om-Ca-L-54, and *V. jasicida* strain Dl-Cu-L-65, were confirmed via qPCR, 16S ribosomal RNA gene sequencing, and MALDI-TOF MS.

**Figure 3:**
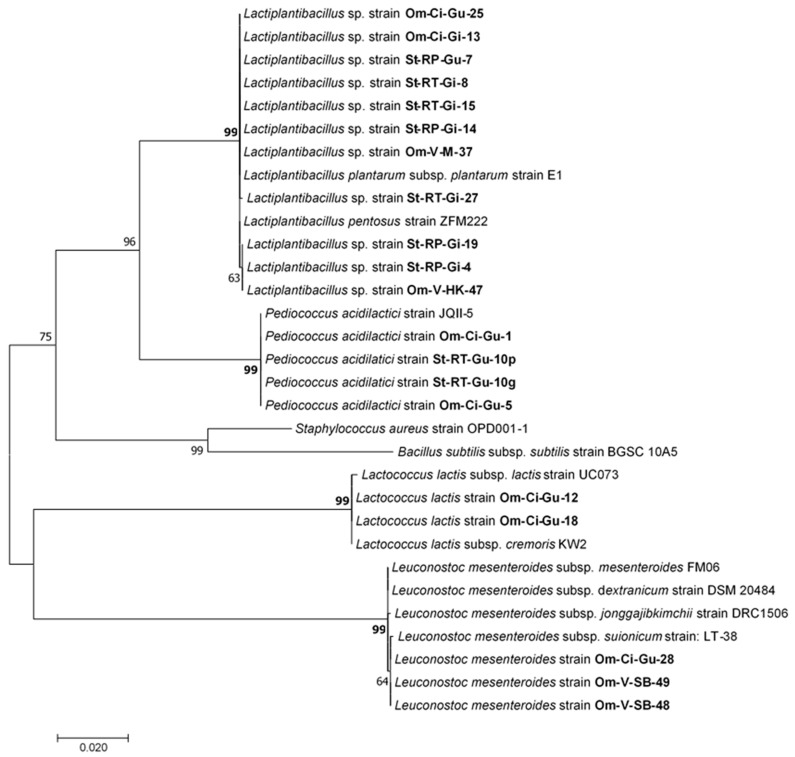
Maximum likelihood phylogenetic original tree based on the Tamura–Nei model. The tree with the highest log likelihood (−4401.58) is shown. The analysis was performed including 31 nucleotide sequences. The percentage of trees in which the associated taxa clustered together is shown next to the branches. The tree is drawn to scale, with branch lengths measured in the number of substitutions per site. All positions containing gaps and missing data were eliminated. There were a total of 1342 positions in the final dataset.

A total of 20 LAB isolates were obtained and identified, which belonged to the genera and species classified as QPS by the EFSA. This included 17 isolates from healthy animals as well as 3 obtained from animals displaying clinical signs of disease. In Figure 3, the maximum likelihood phylogenetic tree illustrates the evolutionary relationships among these LAB strains.

Several of the isolates obtained from individual fish and organs appeared to have almost identical sequences, suggesting that they might be duplicates. For instance, the pair of *P. acidilactici* strains St-RT-Gu-10p and St-RT-Gu-10g isolated from the same fish showed nearly the same sequence. The same observation applies to other pairs: *Lactiplantibacillus* strains St-RP-Gi-19 and St-RP-Gi-4, *Leuconostoc mesenteroides* strains Om-V-SB-48 and Om-V-SB-49, *Lactococcus lactis* strains Om-Ci-Gu-12 and Om-Ci-Gu-18, and *Lactiplantibacillus* strains St-RT-Gi-8 and St-RT-Gi-15. To avoid potential redundancy in the assays due to these likely duplications, only one isolate from each pair was included in the subsequent experiments, resulting in a set of fifteen distinct LAB isolates for further testing.

### 3.2. Antibiotic Susceptibility Test on LAB Probiotic Candidates

The results from the AST are displayed in Table 5 and were interpreted based on the CLSI guidelines as outlined in M45 “Methods for Antimicrobial Dilution and Disk Susceptibility Testing of Infrequently Isolated or Fastidious Bacteria” and CLSI document M100 “Performance Standards for Antimicrobial Susceptibility Testing” [45,46].

In accordance with the CLSI M45 guidelines, the interpretive criteria for *Lactiplantibacillus* sp., *Pediococcus* sp., and *Leuconostoc* sp. were adapted from those established for *Enterococcus* sp., detailed in the CLSI M100 document. Similarly, the criteria for *Lactococcus* sp. were based on those for *Streptococcus* sp. In cases where reference data for specific antibiotics in this species were not available, only the observed inhibition diameters were reported. The bacteria were categorized as resistant (R), intermediate (I), or susceptible (S) based on these criteria.

### 3.3. Antagonistic In Vitro Assay by Agar Plug Diffusion Method

When a 5 µL drop of the probiotic candidate broth culture was placed onto the buffered MRS miniplate before the assay, it formed a dot of approximately 10 mm in diameter. Table 6 displays the inhibition diameters in millimeters. The extent of inhibition on the pathogen’s growth was categorized based on the diameter as “none”, “slight”,” medium”, “high “, and “very high”. If the measured inhibition diameter was equal to the size of the drop, this indicated that inhibition occurred solely at the site of the probiotic drop. However, if the diameter was larger, it signified the presence of an inhibition zone extending beyond the perimeter of the probiotic drop towards the pathogen.

With the exception of the assay against *V. jasicida*, where none of the LAB strains exhibited inhibitory effects, it was observed that the LAB strains were capable of inhibiting the growth of the pathogens to varying degrees.

### 3.4. Antagonistic In Vitro Assay by Agar Well Diffusion Method

The supernatants from the pure LAB cultures, neutralized to a pH of 7, were tested for their inhibitory effect on pathogen growth. Table 7 presents the results, with the inhibition classified as ‘none’, ‘slight’, ‘medium’, ‘high’, and ‘very high’. It was observed that the supernatants did not affect Gram-negative bacteria. However, all the supernatants from the *Lactiplantibacillus* isolates demonstrated inhibitory effects against *C. maltaromaticum* and *V. salmoninarum*. This inhibition suggests the presence of active compounds produced by the LAB that are not sensitive to pH changes. The specific nature of these antimicrobial activities, particularly those evident in the agar well diffusion assays, was not characterized in this study.

### 3.5. Toxicity Assay of Live Bacteria on Embryonic Eggs of Salmo trutta

From the fifteen LAB isolates initially tested *in vitro*, six were selected for the *in vivo* assays: *Lactococcus lactis* Om-Ci-Gu-12, *Leuconostoc mesenteroides* Om-V-SB-48, *P. acidilactici* St-RT-Gu-10p, *Lactiplantibacillus* sp. St-RP-Gi-4, *Lactiplantibacillus* sp. Om-V-HK-47, and *Lactiplantibacillus* sp. Om-Ci-Gi-13. This selection was based on a combination of factors, including the bacterial species, the fish species from which they were isolated, their antibiotic resistance profiles, *in vitro* antagonistic activity against pathogens, and the antagonistic capability of their supernatants. Additionally, a couple of LAB strains isolated from diseased animals were included. This choice was made due to experimental capacity limitations, aiming to representatively encompass the diversity of the initially obtained isolates.

The numbers of dead, living, and hatched eggs on the 16^th^ day after the LAB treatment are presented in Table 8. The normality of the data was individually assessed for each group of replicates within every treatment using the Shapiro–Wilk test. Most groups exhibited a normal distribution for the count of dead and hatched eggs, except for the LAB Om-Ci-Gi-13-treated group. Consequently, group comparisons were conducted utilizing the Kruskal–Wallis test. The resulting *p*-values (<0.001) indicated significant differences among the groups treated with different LAB strains.

**Figure 4:**
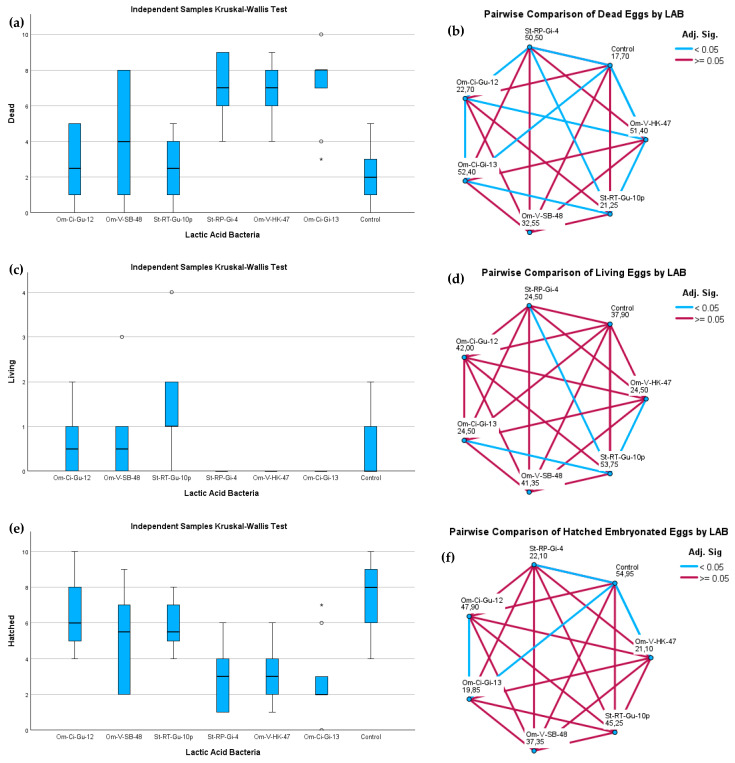
Independent samples Kruskal–Wallis test results are presented in panels (**a**,**c**,**e**) for the mortality, survival, and hatching rates of *S. trutta* eggs, respectively, sixteen days after exposure to LAB strains. Panels (**b**,**d**,**f**) illustrate the pairwise comparison results for dead, living, and hatched eggs, respectively, with each node indicating the average rank of each LAB sample. *Lactiplantibacillus* Om-Ci-Gi-13, Om-V-HK-47, and St-RP-Gi-4 were identified as the LAB strains that induced the highest mortality rates, while *P. acidilactici* St-RT-Gu-10p and *Lactococcus lactis* Om-Ci-Gu-12 demonstrated the lowest mortality rates, closely aligning with the control.

A pairwise analysis involving multiple comparisons among the seven groups was simultaneously performed with the Kruskal–Wallis test, serving as a non-parametric ANOVA, confirming significant differences between the groups. As depicted in Figure 4, the groups treated with *Lactiplantibacillus* strains showed the highest count of dead eggs, with no observation of living eggs; only the larvae from hatched eggs survived. In contrast, the control group and those treated with *P. acidilactici* and *Lactococcus lactis* strains exhibited the lowest count of dead eggs by the end of the assay. In these groups, not only did the larvae from hatched eggs survive but also those from living embryonated eggs survived.

Regarding the group treated with the *Leuconostoc mesenteroides* strain, a notable data dispersion was observed between replicates. Concerning the count of dead eggs, it could be aligned with both the *Lactiplantibacillus* and the *Pediococcus*/*Lactococcus* groups, but it diverged from the *Lactiplantibacillus* groups as it supported not only the survival of larvae from hatched eggs but also that of embryonated eggs.

The measurement of the physicochemical parameters in the water (Table 9) indicated that the levels of oxygen, pH, and conductivity after 24 h of each of the two baths with LAB that were measured provided conditions during immersion that were within an acceptable range for trout eggs. The only exceptionally high value was turbidity, associated with particles, in this case, bacteria, in suspension during the assay. The difference in turbidity between *Lactiplantibacillus* and the other bacteria, given that they were inoculated at the same concentration, could be due to either the larger size of *Lactiplantibacillus* compared to *Pediococcus* and *Lactococcus* or a higher replication rate in the aquatic medium available.

## 4. Discussion

In the present study, strains of *P. acidilactici*, *Lactococcus lactis*, *Lactiplantibacillus* sp., *Leuconostoc mesenteroides*, and *C. divergens* were isolated and identified from the intestinal mucus of healthy *O. mykiss*. From the oral mucus, strains of *Lactiplantibacillus* sp. and *C. maltaromaticum* were obtained and, from the gill mucus, one strain of *Lactiplantibacillus* was recovered. In the case of *S. trutta*, isolates from the intestinal mucus included *Lactiplantibacillus* sp., *P. acidilactici*, and *C. maltaromaticum*; from the oral mucosa, isolates of *C. maltaromaticum* were obtained; from the gill mucus, strains of *Lactiplantibacillus* sp. and *C. maltaromaticum* were isolated; and from the cutaneous mucus, an isolate of *Aerococcus* sp. was found. The literature indicates that potentially probiotic LAB strains have already been isolated and identified from salmonids in other studies. For example, *Lactococcus lactis* has been isolated from the intestinal mucus of Brown trout [14] and from the intestine of Rainbow trout [15,47]; *Lactiplantibacillus plantarum* has been isolated from the intestinal contents of *O. mykiss* [2,15]; *P. acidilactici* has been isolated from Rainbow trout larvae [48] and from the gastrointestinal tract of *S. salar* [49]; and *Leuconostoc mesenteroides* has been isolated from the intestine of Rainbow trout [15]. This is the first study where members belonging to the genus *Lactiplantibacillus* or *P. acidilactici* have been isolated from *S. trutta*.

From an *O. mykiss* presenting with signs of infection, including splenomegaly, swim bladder thickening, and liver adhesion to muscle tissue, a Gram-positive bacterium was initially suspected to be associated with the condition. From these samples, a *Lactiplantibacillus* strain was isolated from the head kidney, and two isolates of *Leuconostoc mesenteroides* were obtained from the swim bladder. Upon review, no prior sequences of these bacteria from these organs were found in GenBank. Regarding pathogenicity, other LAB strains such as *C. maltaromaticum* and *Lactococcus garvieae* are well-documented pathogens associated with infections in fish and also with zoonotic cases in humans [6,7,50,51]. However, for *Lactiplantibacillus plantarum* or *pentosus* or *L. mesenteroides*, we did not find references associated with causing disease in fish. However, the literature reports instances of human infections. For instance, a case of acute acalculous cholecystitis complicated with peritonitis caused by *Lactiplantibacillus plantarum* in a 57-year-old patient [52] and meningoencephalitis caused by *Lactiplantibacillus plantarum* in a 63-year-old man have been reported [53]. There is also a report of *Leuconostoc mesenteroides* causing empyema in a 63-year-old patient [54]. While we cannot definitively claim that the infectious process in this fish was caused by the isolated *Leuconostoc mesenteroides*, as it might be part of the natural microbiota of the swim bladder, the head kidney of fish, being a key component of their immune system, is generally not associated with a significant resident microbiota, so the appearance of this *Lactiplantibacillus* could be considered as an abnormal situation.

Multidrug resistance (MDR) is defined as acquired non-susceptibility to at least one agent in three or more antimicrobial categories, extensive drug resistance (XDR) is defined as non-susceptibility to at least one agent in all but two or fewer antimicrobial categories (i.e., bacterial isolates remain susceptible to only one or two categories), and pandrug resistance (PDR) is defined as non-susceptibility to all agents in all antimicrobial categories [55]. Based solely on the disk assays performed here and the antibiotics tested, *Lactiplantibacillus* does not appear to present antibiotic resistance different from those expected according to the literature and seems safe, but using this definition, isolates of *Lactococcus lactis*, *P. acidilactici*, and *Leuconostoc mesenteroides*, a priori, could fall into the MDR category and, with the exception of the Om-V-SB-48 isolate, would also be XDR to quinolones. While this approach is useful for initial assays, for an adequate interpretation, it would be necessary to conduct studies to establish breakpoints in the classification of antimicrobial resistances in antibiotic disks or MIC for LAB. In the case of the most commonly used antibiotics in freshwater aquaculture for the treatment of infectious diseases, two of the most frequently used ones are florfenicol and oxytetracycline. It is worth noting that none of the probiotic candidate strains showed resistance to these antibiotics, suggesting that there are no acquired resistances. Therefore, the use of these candidates in aquaculture can be considered safe.

The antagonistic capability of LAB is a key factor in their interaction with pathogenic bacteria, and this property can be studied *in vitro* through antagonistic assays against various pathogens of interest. In LAB, this ability is typically attributed to the secretion of antagonistic compounds and/or digestive enzymes that enable them to compete for space and nutrients [56]. The substances produced by these bacteria include organic acids like lactic acid and acetic acid, diacetyl, ethanol, hydrogen peroxide, and bacteriocins [57]. The antagonistic assays revealed that when the LAB isolates obtained were in direct contact with pathogenic agents, whether Gram-positive or -negative, antagonistic activity appeared and was independent of the medium’s pH. However, when only filtered and neutralized supernatant from pure bacterial culture was used, only the filtrates from *Lactiplantibacillus* continued to be effective, but only against *C. maltaromaticum* and *V. salmoninarum*. This is likely because the initiation of antibacterial metabolite production in these LAB requires the stimulus of the pathogen’s presence [58]. In the case of *Lactiplantibacillus*, they may have developed basal mechanisms that produce metabolites to combat *C. maltaromaticum*, a common Gram-positive bacterium in freshwater salmonids and spoiled food, with which they might more commonly compete for ecological niches [59]. Against another tested Gram-positive pathogen, *Lactococcus garvieae*, the *Lactiplantibacillus* supernatant had no effect. One reason could be that since the widespread vaccination against this pathogen in freshwater Rainbow trout farming in Spain, outbreaks are very rare, and the stimulus for *Lactiplantibacillus* to maintain a basal production of molecules against *Lactococcus garvieae* has not been sustained, as producing these compounds involves an energy expenditure. On the other hand, none of the probiotic candidate isolates were able to inhibit the growth of *V. jasicida*, a bacterium from the marine environment. This might be due, from an evolutionary standpoint, to these LAB never having encountered this pathogen and thus not having developed or acquired molecular tools to combat it. It could also simply be that the isolate used has tools to antagonize the LAB.

The study on embryonated trout eggs revealed significant survival differences when treated with various LAB strains, with *Lactiplantibacillus* proving to be the most toxic at the used concentration. Interestingly, in the groups not treated with *Lactiplantibacillus*, live un-hatched embryonated eggs were present at the end of the experiment, whereas in the *Lactiplantibacillus*-treated groups, only hatched larvae survived. *Lactiplantibacillus* sourced from head kidney showed no significant differences from others in its category.

These isolates of *Lactiplantibacillus* would not been recommended for applications in eggs or larval stages of fish due to their toxicity. If used in these early developmental stages, it would be essential to first evaluate whether cell-free supernatants have an adverse effect. However, isolates of *Lactococcus* or *Pediococcus* could be applied as live bacteria, and their ability to modulate the immune response could be studied. On the other hand, *Lactiplantibacillus* could be applied in juvenile or adult fish, either as a probiotic bacteria [60] or as a postbiotic [61].

There have been studies utilizing *O. mykiss* eggs and larvae to create gnotobiotic models for probiotic research [26,28] or to assess the effect of bacteriophages against fish pathogens [27]. Similarly, *Danio rerio* embryonated eggs have been employed to evaluate the effects of LAB [29]. However there is a notable absence of references regarding the use of embryonated *S. trutta* eggs in evaluating the safety of LAB. *S. trutta* is more sensitive than *O. mykiss* to certain contaminants [23], making it a better toxicity indicator for some substances. Furthermore, *S. trutta* may be more effective in observing the variability in responses to contaminants compared to *D. rerio*. The larger size of *S. trutta* larvae facilitates certain experimental manipulations that are more complex in *D. rerio* due to its smaller size. Among the disadvantages of using *S. trutta* is the greater variability in stress responses, a result of the genetic diversity stemming from the use of eggs from wild populations.

One of the main strengths of this work has been the use of a novel approach, testing an animal model that can be valid for evaluating the toxicity of LAB based on the probiotic strain, and whose results can serve as the basis for selecting probiotic strains with an extra degree of safety to continue the research process with more complex assays.

## 5. Conclusions

Combined *in vitro* antimicrobial activity assays using live bacterial patches and pure culture supernatant of LAB indicate that in order to generate an antagonistic effect by LAB against certain pathogens, probiotic bacteria need to be exposed to or stimulated by the presence of such pathogens.

The antimicrobial activity of a probiotic bacterium against certain pathogens might be influenced by previous and continuous exposure to that pathogen, for instance, in a freshwater environment.

The use of certain LAB species in embryonic eggs or vesiculated larvae should preferably consider the application of metabolites or inactivated probiotic bacteria, and in that way, negative effects can be avoided.

Additionally, the role of certain genera of LAB, generally considered safe, should also be explored as potential pathogens in salmonids.

## Figures and Tables

**Table 1:** Bacterial strains, assays, and growth conditions.

Bacteria	Step	Temp. °C	Time and Growth Conditions
Isolation of LAB	Inoculation of samples	20–25	24 h to 48 h in MRS broth
	Isolation of colonies		24 h to 48 h on MRS agar
	Selection/sub-culture of colonies		24 h to 48 h in MRS broth
Isolation of presumptive fish pathogenic bacteria	Inoculation of samples	20–25	16 h to 48 h in TSB, AOE or MRS broth
	Isolation of colonies		16 h to 48 h on TSA, AOE agar or MRS agar
	Selection/sub-culture of colonies		16 h to 48 h in TSB, AOE or MRS broth
*Lactiplantibacillus* sp.	AST	37	24 h in TSB, then 48 h on TSA
	Agar plug diffusion method		24 h in MRS broth, 24 h on buffered MRS agar
	Agar well diffusion method		24 h in MRS broth
*Lactococcus lactis*	AST	37	48 h in TSB, then 48 h on TSA
	Agar plug diffusion method		48 h in MRS broth, 48 h on buffered MRS agar
	Agar well diffusion method		48 h in MRS broth
*Leuconostoc mesenteroides*	AST	30	24 h in TSB, then 48 h on TSA
	Agar plug diffusion method		24 h in MRS broth, 24 h on buffered MRS agar
	Agar well diffusion method		24 h in TSB
*Pediococcus acidilactici*	AST	30	24 h in TSB, then 48 h in TSA
	Agar plug diffusion method		24 h in MRS broth, 24 h on buffered MRS agar
	Agar well diffusion method		24 h in MRS broth
*A. salmonicida* subsp. *salmonicida*	Antagonistic assays	25	24 h in TSB and after the mass inoculation, 24 h in TSA
*Carnobacterium maltaromaticum*	Antagonistic assays	25	24 h in TSB and after the mass inoculation, 24 h in TSA
*Yersinia ruckeri*	Antagonistic assays	25	24 h in TSB and after the mass inoculation, 24 h in TSA
*Vagococcus salmoninarum*	Antagonistic assays	25	48 h in TSB and after the mass inoculation, 48 h in TSA
*Lactococcus garvieae*	Antagonistic assays	37	24 h in TSB and after the mass inoculation, 24 h in TSA
*Vibrio jasicida*	Antagonistic assays	30	24 h in marine broth and after the mass inoculation, 24 h in marine agar

All the steps of isolation, growth and assays were conducted under aerobic conditions.

**Table 2:** Sequences of PCR primers targeting fish pathogens.

Pathogen	Primer	Sequence 5′ to 3′	Amplicon/ Reference
*Flavobacterium columnare*	72 Seghou F	5′-GAAGGAGCTTGTTCCTTT-3′	1260 pb [11]
	1260 Seghou R	5′-GCCTACTTGCGTAGTG-3′	
*Aeromonas salmonicida* subsp. *salmonicida*	aopP Balcázar F	5′-CGGAACGTAATCTGAATTGTTCTTTTC-3′	340 pb [3]
	aopP Balcázar R	5′-ATTGCTTATCGAGGCAGCCAAC-3′	
*C. maltaromaticum*	16S Mohsina F	5′-GAGGGTCATTGGAAACTGGA-3′	219 pb [5]
	16S Mohsina F	5′-CGGAAACCCTCCAACACTTA-3′	
*Vagococcus salmoninarum*	sal Torres F	5′-GACGCTTTCGGGTGTCACTA-3′	543 pb [9]
	sal Torres R	5′-CAGACCAGAGAGTCGCCTTC-3′	
*Yersinia ruckeri*	glnA Keeling F	5′-TCCAGCACCAAATACGAAGG-3′	113 pb [4]
	glnA Keeling R	5′-ACATGGCAGAACGCAGATC-3′	
	glnA Keeling P	HEX-5′-AAGGCGGTTACTTCCCGGTTCC-3′-BHQ1	
*F. psychrophilum*	sig Marancik F	5′-GGTAGCGGAACCGGAAATG-3′	77 pb [8]
	sig Marancik R	5′-TTTCTGCCACCTAGCGAATACC-3′	
	sig Marancik P	FAM-5′-CGCTTCCTGAGCCAGA-3′-BHQ1	
*Lactococcus garvieae*	ITS Chapela F	5′-ACTTTATTCAGTTTTGAGGGGTCT-3′	190 pb [10]
	ITS Chapela R	5′-TTTAACGTCTTCGTTGACCAGA-3′	
	ITS Chapela P	HEX-5′-AGAGAAGGGGCCTTAGCTC-3′-MGB	
*Renibacterium salmoninarum*	RS 1238 Elliot F	5′-GTGACCAACACCCAGATATCCA-3′	69 pb [32]
	RS 1307 Elliot R	5′-TCGCCAGACCACCATTTACC-3′	
	RS 1262 Elliot P	FAM-5′-CACCAGATGGAGCAAC-3′-MGB	
Elongation factor 1α salmonids (Housekeeping gene)	elf-1a GIM-2 F	5′-GCCCCTCCAGGAYGTYTACAA-3′	146 pb [34]
	elf-1a GIM-2 R	5′-CCACACGGCCCACRGGTAC-3′	
	elf-1a GIM-2 P	FAM-5′-ATCGGYGGTATTGGAAC-3′-MGB	

**Table 3:** Bacterial species strains used for the phylogenetic analysis.

Species/Strain	Genbank Accession No.	Genomic Nucleotide Position
*Lactococcus lactis* subsp. *cremoris* KW2	CP004884.1	500179–502418
*Leuconostoc mesenteroides* subsp. *dextranicum* strain DSM 20484	CP012009.1	374968–377287
*Leuconostoc mesenteroides* subsp. *jonggajibkimchii* strain DRC1506	CP014611.1	22983–25302
*Leuconostoc mesenteroides* subsp. *mesenteroides* FM06	CP020731.1	718521–720840
*Leuconostoc mesenteroides* subsp. *suionicum* strain LT-38	AP017935.1	270279–272598
*Pediococcus acidilactici* strain JQII-5	CP023654.1	219495–221665
*Lactiplantibacillus plantarum* subsp. *plantarum* strain E1	CP031771.1	2889706–2891876
*Lactiplantibacillus pentosus* strain ZFM222	CP032654.1	1589825–1591995
*Lactococcus lactis* subsp. *lactis* strain UC073	CP068698.2	429176–431415
*Bacillus subtilis* subsp. *subtilis* strain BGSC 10A5	CP101936.1	184980–187588
*Staphylococcus aureus* strain OPD001-1	CP121234.1	635770–638640

**Table 4:** LAB and pathogens obtained and/or identified in this study.

Host	Isolation Source	Species/Strain	pb.	Genbank Accession No.
*O. mykiss*	Intestinal mucus	*P. acidilactici* strain Om-Ci-Gu-1	2160	OR734325
*O. mykiss*	Intestinal mucus	*P. acidilactici* strain Om-Ci-Gu-5	1580	OR734326
*O. mykiss*	Intestinal mucus	*Lactococcus lactis* strain Om-Ci-Gu-12	1354	OR734327
*O. mykiss*	Intestinal mucus	*Lactococcus lactis* strain Om-Ci-Gu-18	2241	OR734328
*O. mykiss*	Intestinal mucus	*Lactiplantibacillus* sp. strain Om-Ci-Gu-25	2171	OR734329
*O. mykiss*	Intestinal mucus	*Leuconostoc mesenteroides* strain Om-Ci-Gu-28	1364	OR734330
*O. mykiss*	Intestinal mucus	*C. divergens* Om-V-Gu-34	2203	OR753895
*O. mykiss*	Mouth mucus	*Lactiplantibacillus* sp. strain Om-V-M-37	1376	OR734332
*O. mykiss*	Mouth mucus	*C. maltaromaticum* strain Om-Ca-M-31	2373	OR753882
*O. mykiss*	Mouth mucus	*C. maltaromaticum* strain Om-Ca-M-36c1	2365	OR753883
*O. mykiss*	Mouth mucus	*C. maltaromaticum* strain Om-Ca-M-36c2	2355	OR753884
*O. mykiss*	Gills mucus	*Lactiplantibacillus* sp. strain Om-Ci-Gi-13	1587	OR734324
*S. trutta*	Intestinal mucus	*Lactiplantibacillus* sp. strain St-RP-Gu-7	1560	OR734338
*S. trutta*	Intestinal mucus	*P. acidilactici* strain St-RT-Gu-10g	2171	OR734342
*S. trutta*	Intestinal mucus	*P. acidilactici* strain St-RT-Gu-10p	1580	OR734343
*S. trutta*	Intestinal mucus	*C. maltaromaticum* strain St-BC-Gu-33	2359	OR753887
*S. trutta*	Intestinal mucus	*C. maltaromaticum* strain St-PS-Gu-41	2332	OR753889
*S. trutta*	Mouth mucus	*C. maltaromaticum* strain St-Ba-M-35	2366	OR753885
*S. trutta*	Mouth mucus	*C. maltaromaticum* strain St-Ba-M-42	2371	OR753886
*S. trutta*	Gills mucus	*Lactiplantibacillus* sp. strain St-RP-Gi-4	1561	OR734335
*S. trutta*	Gills mucus	*Lactiplantibacillus* sp. strain St-RP-Gi-14	2165	OR734336
*S. trutta*	Gills mucus	*Lactiplantibacillus* sp. strain St-RP-Gi-19	2172	OR734337
*S. trutta*	Gills mucus	*Lactiplantibacillus* sp. strain St-RT-Gi-8	1571	OR734339
*S. trutta*	Gills mucus	*Lactiplantibacillus* sp. strain St-RT-Gi-15	2144	OR734340
*S. trutta*	Gills mucus	*Lactiplantibacillus* sp. strain St-RT-Gi-27	1569	OR734341
*S. trutta*	Gills mucus	*C. maltaromaticum* strain St-RP-Gi-2	2320	OR753890
*S. trutta*	Gills mucus	*C. maltaromaticum* strain St-RP-Gi-9	2323	OR753891
*S. trutta*	Gills mucus	*C. maltaromaticum* strain St-RP-Gi-24	2348	OR753892
*S. trutta*	Gills mucus	*C. maltaromaticum* strain St-RT-Gi-26p	2336	OR753896
*S. trutta*	Skin mucus	*Aerococcus* sp. strain St-BC-SK-82	2230	OR753888
*O. mykiss*	Liver	*Y. ruckeri* strain Om-Ca-L-54	2497	OR763346
*O. mykiss*	Liver	*V. salmoninarum* strain Om-V-L-69	1419	OR763343
*O. mykiss*	Head kidney	*Lactiplantibacillus* sp. strain Om-V-HK-47	1600	OR734331
*O. mykiss*	Head kidney	*Lactococcus garvieae* strain Om-Pe-HK-61	2320	OR763345
*O. mykiss*	Swim bladder	*Leuconostoc mesenteroides* strain Om-V-SB-48	1620	OR734333
*O. mykiss*	Swim bladder	*Leuconostoc mesenteroides* strain Om-V-SB-49	2320	OR734334
*D. labrax*	Liver	*V. jasicida* strain Dl-Cu-L-65	1637	OR763344
*S. trutta*	Head kidney	*C. maltaromaticum* strain St-PS-HK-63	1609	OR753893
*S. trutta*	Head kidney	*C. maltaromaticum* strain St-PS-HK-64	1642	OR753894

The 16S rRNA gene was sequenced for all the isolates listed in this table. For some isolates, a contiguous sequence of over 2000 nucleotides, which includes the 16S rRNA gene, the ITS-1 region, and a fragment of the 23S rRNA gene, was successfully assembled.

**Table 5:** Antimicrobial susceptibility test results of the LAB probiotic candidates.

Antimicrobial Agent		*Lactococcus lactis* Om-Ci-Gu-12	*Leuconostoc mesenteroides* Om-Ci-Gu-28	*Leuconostoc mesenteroides* Om-V-SB-48	*P. acidilactici* Om-Ci-GU-1	*P. acidilactici* Om-Ci-GU-5	*P. acidilactici* St-RT-Gu-10p	*Lactiplantibacillus* sp. St-RP-Gi-4	*Lactiplantibacillus* sp. St-RT-Gi-27	*Lactiplantibacillus* sp. Om-V-HK-47	*Lactiplantibacillus* sp. St-RP-Gu-7	*Lactiplantibacillus* sp. Om-Ci-Gu-25	*Lactiplantibacillus* sp. Om-V-M-37	*Lactiplantibacillus* sp. St-RP-Gi-14	*Lactiplantibacillus* sp. St-RT-Gi-8	*Lactiplantibacillus* sp. Om-Ci-Gi-13
Glycopeptides	Vancomycin	0 ^R^	0 ^R^	0 ^R^	0 ^R^	0 ^R^	0 ^R^	0 ^R^	0 ^R^	0 ^R^	0 ^R^	0 ^R^	0 ^R^	0 ^R^	0 ^R^	0 ^R^
Penicillins	Amoxicillin/Clavulanic	20	20	18	20	20	21	22	26	23	22	24	36	21	25	29
	Penicillin G	27 ^S^	23 ^S^	25 ^S^	27 ^S^	25 ^S^	26 ^S^	0 ^R^	0 ^R^	0 ^R^	0 ^R^	0 ^R^	26 ^S^	0 ^R^	0 ^R^	0 ^R^
	Ampicillin	12 ^R^	23 ^S^	15 ^R^	13 ^R^	12 ^R^	13 ^R^	12 ^R^	15 ^R^	16 ^R^	15 ^R^	12 ^R^	33 ^S^	23 ^S^	18 ^S^	17 ^S^
	Oxacillin	0	0	0	0	0	0	0	0	0	0	0	0	0	0	0
Aminoglycosides	Kanamycin	0	11	20	11	11	11	17	16	16	18	16	24	17	19	18
	Neomycin	18	16	25	16	16	17	20	22	19	21	23	27	20	23	22
	Streptomycin	9	8	19	9	10	9	19	23	23	22	25	26	15	24	23
	Gentamicin	18	14	24	15	16	14	21	19	21	20	22	27	18	23	21
Dihydrofolate reduc.	Trimethoprim	0	0	0	0	0	0	23	16	14	22	25	20	0	16	16
Macrolides	Erythromycin	33 ^S^	40 ^S^	35 ^S^	44 ^S^	35 ^S^	44 ^S^	37 ^S^	36 ^S^	35 ^S^	43 ^S^	47 ^S^	35 ^S^	43 ^S^	32 ^S^	35 ^S^
Cephalosporins	Cefuroxime	17	17	16	18	20	19	33	31	21	22	29	28	26	20	25
	Ceftriaxone	0 ^R^	0	0	0	0	0	38	35	23	26	35	36	33	20	26
Tetracyclines	Tetracycline	20 ^S^	17^I^	28 ^S^	20 ^S^	19 ^S^	21 ^S^	22 ^S^	21 ^S^	19 ^S^	22 ^S^	22 ^S^	23 ^S^	22 ^S^	18^I^	19 ^S^
	Doxycycline	21 ^S^	20 ^S^	30 ^S^	26 ^S^	25 ^S^	24 ^S^	27 ^S^	27 ^S^	22 ^S^	24 ^S^	27 ^S^	23 ^S^	25 ^S^	19 ^S^	21 ^S^
	Oxytetracycline	19	19	30	23	20	22	25	23	21	23	25	22	22	20	21
Lincosamides	Clindamycin	33	32	32	35	32	34	26	17	12	14	17	19	12	13	13
Quinolones	Nalidixic Acid	0	0	0	0	0	0	0	0	0	0	0	0	0	0	0
	Flumequine	0	0	0	0	0	0	15	15	14	13	14	15	12	14	13
	Ciprofloxacin	0 ^R^	0 ^R^	19 ^I^	0 ^R^	0 ^R^	0 ^R^	14 ^R^	9 ^R^	9 ^R^	18 ^I^	21 ^S^	15 ^R^	16 ^I^	10 ^R^	12 ^R^
Nitrofurans	Nitrofurantoine	23 ^S^	21 ^S^	0 ^R^	20 ^S^	22 ^S^	22 ^S^	31 ^S^	36 ^S^	18 ^S^	37 ^S^	31 ^S^	25 ^S^	33 ^S^	17 ^S^	19 ^S^
Amphenicols	Florfenicol	30 ^S^	33 ^S^	35 ^S^	32 ^S^	30 ^S^	28 ^S^	32 ^S^	34 ^S^	33 ^S^	36 ^S^	35 ^S^	30 ^S^	30 ^S^	33 ^S^	34 ^S^
	Chloramphenicol	27 ^S^	25 ^S^	30 ^S^	25 ^S^	26 ^S^	30 ^S^	36 ^S^	32 ^S^	29 ^S^	33 ^S^	35 ^S^	28 ^S^	30 ^S^	25 ^S^	27 ^S^

The results are expressed as the diameter of inhibition in millimeters. The bacteria were categorized as resistant (^R^), intermediate (^I^), or susceptible (^S^). Gray shading in the inhibition zones on the table indicates bacterial resistance to the tested antibiotic, as per the criteria or if the antibiotic failed to inhibit the growth of the LAB entirely.

**Table 6:** Antagonistic *in vitro* assay by agar plug diffusion method.

Pathogen				*Lactococcus lactis* Om-Ci-Gu-12	*Leuconostoc mesenteroides* Om-Ci-Gu-28	*Leuconostoc mesenteroides* Om-V-SB-48	*P. acidilactici* Om-Ci-GU-1	*P. acidilactici* Om-Ci-GU-5	*P. acidilactici* St-RT-Gu-10p	*Lactiplantibacillus* sp. St-RP-Gi-4	*Lactiplantibacillus* sp. St-RT-Gi-27	*Lactiplantibacillus* sp. Om-V-HK-47	*Lactiplantibacillus* sp. St-RP-Gu-7	*Lactiplantibacillus* sp. Om-Ci-Gu-25	*Lactiplantibacillus* sp. Om-V-M-37	*Lactiplantibacillus* sp. St-RP-Gi-14	*Lactiplantibacillus* sp. St-RT-Gi-8	*Lactiplantibacillus* sp. Om-Ci-Gi-13
*C. maltaromaticum* St-PS-HK-63				10	11	8	14	0	11	12	10	11	11	12	10	12	12	12
*V. salmoninarum* Om-V-L-69				13	20	16	15	13	13	17	17	21	17	19	21	21	22	30
*Lactococcus garvieae* Om-Pe-HK-61				10	11	5	7	6	8	7	10	0	0	10	7	11	8	13
*Y. ruckeri* Om-Ca-L-54				12	0	0	9	14	13	8	11	15	14	11	10	9	11	15
*V. jasicida* Dl-Cu-L-65				0	0	0	0	0	0	0	0	0	0	0	0	0	0	0
*A. salmonicida* subsp. *salmonicida* St-Mu-Sk				32	16	0	12	19	27	29	20	26	12	30	50	32	18	32
	None (0 mm)		Slight (1 ≤ 10 mm)		Medium (11 ≤ 20 mm)					High (21–30 mm)				Very high (>30 mm)				

The results are expressed as the diameter of inhibition in millimeters.

**Table 7:** Antagonistic *in vitro* assay by agar well diffusion method.

Pathogen				*Lactococcus lactis* Om-Ci-Gu-12	*Leuconostoc mesenteroides* Om-Ci-Gu-28	*Leuconostoc mesenteroides* Om-V-SB-48	*P. acidilactici* Om-Ci-GU-1	*P. acidilactici* Om-Ci-GU-5	*P. acidilactici* St-RT-Gu-10p	*Lactiplantibacillus* sp. St-RP-Gi-4	*Lactiplantibacillus* sp. St-RT-Gi-27	*Lactiplantibacillus* sp. Om-V-HK-47	*Lactiplantibacillus* sp. St-RP-Gu-7	*Lactiplantibacillus* sp. Om-Ci-Gu-25	*Lactiplantibacillus* sp. Om-V-M-37	*Lactiplantibacillus* sp. St-RP-Gi-14	*Lactiplantibacillus* sp. St-RT-Gi-8	*Lactiplantibacillus* sp. Om-Ci-Gi-13
*C. maltaromaticum* St-PS-HK-63				0	0	0	0	0	0	5	5	5	5	5	6	4	3	5
*V. salmoninarum* Om-V-L-69				0	0	0	0	0	0	5	7	6	7	6	7	5	5	5
*Lactococcus garvieae* Om-Pe-HK-61				0	0	0	0	0	0	0	0	0	0	0	0	0	0	0
*Y. ruckeri* Om-Ca-L-54				0	0	0	0	0	0	0	0	0	0	0	0	0	0	0
*V. jasicida* Dl-Cu-L-65				0	0	0	0	0	0	0	0	0	0	0	0	0	0	0
*A. salmonicida* subsp. *salmonicida* St-Mu-Sk				0	0	0	0	0	0	0	0	0	0	0	0	0	0	0
	None (<1 mm)		Slight (1 ≤ 3 mm)		Medium (4 ≤ 6 mm)					High (7 ≤ 9 mm)				Very high (>10 mm)				

The results are expressed as the radius of inhibition in millimeters.

**Table 8:** Counting of dead, living, and hatched eggs.

Flask/Replicate	Assessment of Fish Eggs	*Lactococcus lactis* Om-Ci-Gu-12	*Leuconostoc mesenteroides* Om-V-SB-48	*P. acidilactici* St-RT-Gu-10p	*Lactiplantibacillus* sp. St-RP-Gi-4	*Lactiplantibacillus* sp. Om-V-HK-47	*Lactiplantibacillus* sp. Om-Ci-Gi-13	Control
1	Dead	5	1	1	6	7	8	1
	Living	0	1	2	0	0	0	0
	Hatched	5	8	7	4	3	2	9
2	Dead	5	0	0	7	6	8	2
	Living	1	1	4	0	0	0	0
	Hatched	4	9	6	3	4	2	8
3	Dead	4	1	0	4	9	4	0
	Living	2	3	2	0	0	0	0
	Hatched	4	6	8	6	1	6	10
4	Dead	1	2	4	9	4	7	5
	Living	0	1	1	0	0	0	1
	Hatched	9	7	5	1	6	3	4
5	Dead	2	8	2	9	7	3	1
	Living	1	0	1	0	0	0	0
	Hatched	7	2	7	1	3	7	9
6	Dead	1	5	3	7	8	8	2
	Living	1	0	2	0	0	0	0
	Hatched	8	5	5	3	2	2	8
7	Dead	5	6	5	9	5	8	1
	Living	0	0	1	0	0	0	1
	Hatched	5	4	4	1	5	2	8
8	Dead	0	3	2	6	6	7	4
	Living	0	1	0	0	0	0	1
	Hatched	10	6	8	4	4	3	5
9	Dead	2	8	4	4	9	10	3
	Living	0	0	1	0	0	0	0
	Hatched	8	2	5	6	1	0	7
10	Dead	3	8	5	7	8	8	2
	Living	2	0	0	0	0	0	2
	Hatched	5	2	5	3	2	2	6

Sixteen days after the initial exposure to the LABs, at 448 dd post fertilization, the experiment concluded with the assessment of the quantity of deceased and viable eggs, along with the count of hatched live larvae.

**Table 9:** Table of water quality parameters.

Day of Assay	Parameter	*Lactococcus lactis* Om-Ci-Gu-12	*Leuconostoc mesenteroides* Om-V-SB-48	*P. acidilactici* St-RT-Gu-10p	*Lactiplantibacillus* sp. St-RP-Gi-4	*Lactiplantibacillus* sp. Om-V-HK-47	*Lactiplantibacillus* sp. Om-Ci-Gi-13	Control
24 h after 1st bath	O_2_%	106.1	108.7	93.2	96.0	81.2	95.5	97.9
	O_2_ (mg/mL)	10.4	10.2	8.7	8.9	7.6	9.0	9.4
	pH	7.6	7.5	7.5	7.6	7.4	7.6	7.4
	Conductivity	2.4	2.3	2.4	2.3	2.4	2.3	1.6
	Turbidity (NTU)	102	209	124	414	598	493	0.74
24 h after 2nd bath	O_2_%	84.8	87.4	86.1	73.4	83.0	72.2	93.8
	O_2_ mg/mL	9.1	9.6	8.2	7.0	9.4	6.82	9.0
	pH	7.6	7.6	7.8	7.8	7.8	7.55	7.9
	Conductivity	2.5	2.6	2.6	2.7	2.6	2.78	1.7
	Turbidity (NTU)	209	289	272	586	468	571	0.77

This table presents the measured physicochemical parameters of water. The levels of dissolved oxygen, pH, and conductivity were within generally acceptable ranges for the development of the eggs. However, significant variations in turbidity were observed, with some exceptionally high values.

## Data Availability

This study has contributed 16S rRNA sequences from various bacteria to the GenBank database. These sequences, used in constructing a phylogenetic tree along with reference sequences from GenBank, are publicly available for consultation and use in research. The specific details and GenBank accession numbers for these sequences can be found in the Results section of this article.

## References

[1] Aquaculture Business Association of Spain (APROMAR) Aquaculture in Spain 2023 APROMAR 2023. https://apromar.es/wp-content/uploads/2023/10/Aquaculture_in_Spain_2023_APROMAR.pdf.

[2] Fečkaninová A., Koščová J., Mudroňová D., Schusterová P., Cingeľová Maruščáková I., Popelka P. (2019). Characterization of Two Novel Lactic Acid Bacteria Isolated from the Intestine of Rainbow Trout (Oncorhynchus Mykiss, Walbaum) in Slovakia. Aquaculture.

[3] Balcázar J.L., Vendrell D., De Blas I., Ruiz-Zarzuela I., Gironés O., Múzquiz J.L. (2007). Quantitative Detection of Aeromonas Salmonicida in Fish Tissue by Real-Time PCR Using Self-Quenched, Fluorogenic Primers. J. Med. Microbiol..

[4] Keeling S.E., Johnston C., Wallis R., Brosnahan C.L., Gudkovs N., McDonald W.L. (2012). Development and Validation of Real-Time PCR for the Detection of Yersinia Ruckeri: Yersinia Ruckeri Real-Time PCR. J. Fish Dis..

[5] Mohsina K., Kaur M., Bowman J.P., Powell S., Tamplin M.L. (2020). qPCR Quantification of Carnobacterium Maltaromaticum, Brochothrix Thermosphacta, and Serratia Liquefaciens Growth Kinetics in Mixed Culture. J. Microbiol. Methods.

[6] Raza S., Koh Y., Yoon S.-S., Woo S.-Y., Ahn K.-S., Kim H.-L., Kim H.-N. (2023). Identification of Novel Carnobacterium Maltaromaticum Strains in Bone Marrow Samples of Patients with Acute Myeloid Leukemia Using a Metagenomic Binning Approach. Int. Microbiol..

[7] Smith S.A., Newman S.J., Harrison C.E., Loch T.P. (2023). First Isolation of *Carnobacterium Maltaromaticum* from Farmed Rainbow Trout in Virginia. J. Aquat. Anim. Health.

[8] Marancik D.P., Wiens G.D. (2013). A Real-Time Polymerase Chain Reaction Assay for Identification and Quantification of *Flavobacterium Psychrophilum* and Application to Disease Resistance Studies in Selectively Bred Rainbow Trout *Oncorhynchus Mykiss*. FEMS Microbiol. Lett..

[9] Torres-Corral Y., Fernández-Álvarez C., Santos Y. (2019). High-throughput Identification and Quantification of *Vagococcus Salmoninarum* by SYBR Green I-based Real-time PCR Combined with Melting Curve Analysis. J. Fish Dis..

[10] Chapela M.-J., Ferreira M., Varela C., Arregui L., Garrido-Maestu A. (2018). Development of a Multiplex Real-Time PCR Method for Early Diagnosis of Three Bacterial Diseases in Fish: A Real-Case Study in Trout Aquaculture. Aquaculture.

[11] Seghouani H., Garcia-Rangel C.-E., Füller J., Gauthier J., Derome N. (2017). Walleye Autochthonous Bacteria as Promising Probiotic Candidates against Flavobacterium Columnare. Front. Microbiol..

[12] Bondad-Reantaso M.G., MacKinnon B., Karunasagar I., Fridman S., Alday-Sanz V., Brun E., Le Groumellec M., Li A., Surachetpong W., Karunasagar I. (2023). Review of Alternatives to Antibiotic Use in Aquaculture. Rev. Aquac..

[13] Pérez-Sánchez T., Mora-Sánchez B., Balcázar J.L. (2018). Biological Approaches for Disease Control in Aquaculture: Advantages, Limitations and Challenges. Trends Microbiol..

[14] Pérez T., Balcázar J.L., Peix A., Valverde A., Velázquez E., de Blas I., Ruiz-Zarzuela I. (2011). Lactococcus Lactis Subsp. Tructae Subsp. Nov. Isolated from the Intestinal Mucus of Brown Trout (Salmo Trutta) and Rainbow Trout (Oncorhynchus Mykiss). Int. J. Syst. Evol. Microbiol..

[15] Pérez-Sánchez T., Balcázar J.L., García Y., Halaihel N., Vendrell D., de Blas I., Merrifield D.L., Ruiz-Zarzuela I. (2011). Identification and Characterization of Lactic Acid Bacteria Isolated from Rainbow Trout, Oncorhynchus Mykiss (Walbaum), with Inhibitory Activity against Lactococcus Garvieae: Trout Endogenous LAB Antagonise L. Garvieae. J. Fish Dis..

[16] Abid A., Davies S.J., Waines P., Emery M., Castex M., Gioacchini G., Carnevali O., Bickerdike R., Romero J., Merrifield D.L. (2013). Dietary Synbiotic Application Modulates Atlantic Salmon (Salmo Salar) Intestinal Microbial Communities and Intestinal Immunity. Fish Shellfish Immunol..

[17] Koutsoumanis K., Allende A., Alvarez-Ordóñez A., Bolton D., Bover-Cid S., Chemaly M., De Cesare A., Hilbert F., Lindqvist R., EFSA Panel on Biological Hazards (BIOHAZ) (2023). Statement on How to Interpret the QPS Qualification on ‘Acquired Antimicrobial Resistance Genes’. EFS2.

[18] Koutsoumanis K., Allende A., Alvarez-Ordóñez A., Bolton D., Bover-Cid S., Chemaly M., Davies R., De Cesare A., Hilbert F., EFSA Panel on Biological Hazards (BIOHAZ) (2021). Update of the List of QPS-recommended Biological Agents Intentionally Added to Food or Feed as Notified to EFSA 13: Suitability of Taxonomic Units Notified to EFSA until September 2020. EFS2.

[19] Rychen G., Aquilina G., Azimonti G., Bampidis V., de Lourdes Bastos M., Bories G., Chesson A., Cocconcelli P.S., Flachowsky G., EFSA Panel on Additives and Products or Substances Used in Animal Feed (FEEDAP) (2018). Guidance on the Characterisation of Microorganisms Used as Feed Additives or as Production Organisms. EFS2.

[20] Rychen G., Aquilina G., Azimonti G., Bampidis V., de Lourdes Bastos M., Bories G., Chesson A., Cocconcelli P.S., Flachowsky G., EFSA Panel on Additives and Products or Substances Used in Animal Feed (FEEDAP) (2017). Guidance on the Assessment of the Safety of Feed Additives for the Target Species. EFS2.

[21] Rychen G., Aquilina G., Azimonti G., Bampidis V., de Lourdes Bastos M., Bories G., Chesson A., Cocconcelli P.S., Flachowsky G., EFSA Panel on Additives and Products or Substances Used in Animal Feed (FEEDAP) (2017). Guidance on the Identity, Characterisation and Conditions of Use of Feed Additives. EFS2.

[22] Miller J.L., Miller M.J., De Vlaming V., Larsen K., Smith E., Reece K. (2009). Selection and Application of a Rainbow Trout Toxicity Testing Procedure for Screening Sacramento River Watershed, California Samples. Env. Monit. Assess..

[23] Rigaud C., Härme J., Vehniäinen E.-R. (2022). Salmo Trutta Is More Sensitive than Oncorhynchus Mykiss to Early-Life Stage Exposure to Retene. Comp. Biochem. Physiol. Part C Toxicol. Pharmacol..

[24] Hoitsy G., András W., Thomas M.-P. (2012). Guide to the Small Scale Artificial Propagation of Trout.

[25] Wilkins L.G.E., Rogivue A., Schütz F., Fumagalli L., Wedekind C. (2015). Increased Diversity of Egg-Associated Bacteria on Brown Trout (Salmo Trutta) at Elevated Temperatures. Sci. Rep..

[26] Jimenez Reyes M., Yany G., Romero J. (2017). Protocolo para obtencion de alevines axenicos de trucha arcoiris (*Oncorhynchus mykiss*). Lajar.

[27] Donati V.L., Dalsgaard I., Runtuvuori-Salmela A., Kunttu H., Jørgensen J., Castillo D., Sundberg L.-R., Middelboe M., Madsen L. (2021). Interactions between Rainbow Trout Eyed Eggs and Flavobacterium Spp. Using a Bath Challenge Model: Preliminary Evaluation of Bacteriophages as Pathogen Control Agents. Microorganisms.

[28] Pérez-Pascual D., Vendrell-Fernández S., Audrain B., Bernal-Bayard J., Patiño-Navarrete R., Petit V., Rigaudeau D., Ghigo J.-M. (2021). Gnotobiotic Rainbow Trout (Oncorhynchus Mykiss) Model Reveals Endogenous Bacteria That Protect against Flavobacterium Columnare Infection. PLoS Pathog..

[29] Padeniya U., Larson E.T., Septriani S., Pataueg A., Kafui A.R., Hasan E., Mmaduakonam O.S., Kim G., Kiddane A.T., Brown C.L. (2022). Probiotic Treatment Enhances Pre-feeding Larval Development and Early Survival in Zebrafish *Danio Rerio*. J. Aquat. Anim. Health.

[30] Aquatic Animal Health & Vaccines Centre of Excellence (2017). Field Sampling of Fish for Disease Investigation and Health Monitoring, Guidelines and Procedures Manual.

[31] Chase D.M., Elliott D.G., Pascho R.J. (2006). Detection and Quantification of *Renibacterium Salmoninarum* DNA in Salmonid Tissues by Real-Time Quantitative Polymerase Chain Reaction Analysis. J. VET Diagn. Investig..

[32] Elliott D.G., Applegate L.J., Murray A.L., Purcell M.K., McKibben C.L. (2013). Bench-Top Validation Testing of Selected Immunological and Molecular *Renibacterium Salmoninarum* Diagnostic Assays by Comparison with Quantitative Bacteriological Culture. J. Fish Dis..

[33] Bartkova S., Kokotovic B., Skall H.F., Lorenzen N., Dalsgaard I. (2017). Detection and Quantification of *Aeromonas Salmonicida* in Fish Tissue by Real-Time PCR. J. Fish Dis..

[34] Sepúlveda D., Bohle H., Labra Á., Grothusen H., Marshall S.H. (2013). Design and Evaluation of a Unique RT-qPCR Assay for Diagnostic Quality Control Assessment That Is Applicable to Pathogen Detection in Three Species of Salmonid Fish. BMC Vet. Res..

[35] Weisburg W.G., Barns S.M., Pelletier D.A., Lane D.J. (1991). 16S Ribosomal DNA Amplification for Phylogenetic Study. J. Bacteriol..

[36] Yoon S.-H., Ha S.-M., Kwon S., Lim J., Kim Y., Seo H., Chun J. (2017). Introducing EzBioCloud: A Taxonomically United Database of 16S rRNA Gene Sequences and Whole-Genome Assemblies. Int. J. Syst. Evol. Microbiol..

[37] Kumar S., Tamura K., Nei M. (1994). MEGA: Molecular Evolutionary Genetics Analysis Software for Microcomputers. Bioinformatics.

[38] Kumar S., Stecher G., Tamura K. (2016). MEGA7: Molecular Evolutionary Genetics Analysis Version 7.0 for Bigger Datasets. Mol. Biol. Evol..

[39] Tamura K., Peterson D., Peterson N., Stecher G., Nei M., Kumar S. (2011). MEGA5: Molecular Evolutionary Genetics Analysis Using Maximum Likelihood, Evolutionary Distance, and Maximum Parsimony Methods. Mol. Biol. Evol..

[40] Bouzaine T., Dauphin R.D., Thonart P., Urdaci M.C., Hamdi M. (2005). Adherence and Colonization Properties of Lactobacillus Rhamnosus TB1, a Broiler Chicken Isolate. Lett. Appl. Microbiol..

[41] Li T., Teng D., Mao R., Hao Y., Wang X., Wang J. (2020). A Critical Review of Antibiotic Resistance in Probiotic Bacteria. Food Res. Int..

[42] Anokyewaa M.A., Amoah K., Li Y., Lu Y., Kuebutornye F.K.A., Asiedu B., Seidu I. (2021). Prevalence of Virulence Genes and Antibiotic Susceptibility of Bacillus Used in Commercial Aquaculture Probiotics in China. Aquac. Rep..

[43] Elleuch L., Shaaban M., Smaoui S., Mellouli L., Karray-Rebai I., Fourati-Ben Fguira L., Shaaban K.A., Laatsch H. (2010). Bioactive Secondary Metabolites from a New Terrestrial Streptomyces Sp. TN262. Appl. Biochem. Biotechnol..

[44] Balouiri M., Sadiki M., Ibnsouda S.K. (2016). Methods for *in Vitro* Evaluating Antimicrobial Activity: A Review. J. Pharm. Anal..

[45] Hindler J.A., Richter S.S. (2016). Methods for Antimicrobial Dilution and Disk Susceptibility Testing of Infrequently Isolated or Fastidious Bacteria: M45.

[46] James L.S. (2022). M100 Performance Standards for Antimicrobial.

[47] Araújo C., Muñoz-Atienza E., Pérez-Sánchez T., Poeta P., Igrejas G., Hernández P.E., Herranz C., Ruiz-Zarzuela I., Cintas L.M. (2015). Nisin Z Production by Lactococcus Lactis Subsp. Cremoris WA2-67 of Aquatic Origin as a Defense Mechanism to Protect Rainbow Trout (Oncorhynchus Mykiss, Walbaum) Against Lactococcus Garvieae. Mar. Biotechnol..

[48] Araújo C., Muñoz-Atienza E., Poeta P., Igrejas G., Hernández P., Herranz C., Cintas L. (2016). Characterization of Pediococcus Acidilactici Strains Isolated from Rainbow Trout (Oncorhynchus Mykiss) Feed and Larvae: Safety, DNA Fingerprinting, and Bacteriocinogenicity. Dis. Aquat. Org..

[49] Amin M., Adams M., Bolch C.J.S., Burke C.M. (2017). *In Vitro* Screening of Lactic Acid Bacteria Isolated from Gastrointestinal Tract of Atlantic Salmon (Salmo Salar) as Probiont Candidates. Aquacult. Int..

[50] Meyburgh C., Bragg R., Boucher C. (2017). Lactococcus Garvieae: An Emerging Bacterial Pathogen of Fish. Dis. Aquat. Org..

[51] Rösch R.M., Buschmann K., Brendel L., Schwanz T., Vahl C.-F. (2019). *Lactococcus Garvieae* Endocarditis in a Prosthetic Aortic Valve: A Case Report and Literature Review. J. Investig. Med. High Impact Case Rep..

[52] Tena D., Martínez N.M., Losa C., Fernández C., Medina M.J., Sáez-Nieto J.A. (2013). Acute Acalculous Cholecystitis Complicated with Peritonitis Caused by Lactobacillus Plantarum. Diagn. Microbiol. Infect. Dis..

[53] Biesiada G., Krycińska R., Czepiel J., Stażyk K., Kędzierska J., Garlicki A. (2019). Meningoencephalitis Caused by *Lactobacillus Plantarum*—Case Report. Int. J. Neurosci..

[54] Usta-Atmaca H., Akbas F., Karagoz Y., Piskinpasa M.E. (2015). A Rarely Seen Cause for Empyema: Leuconostoc Mesenteroıdes. J. Infect. Dev. Ctries.

[55] Magiorakos A.-P., Srinivasan A., Carey R.B., Carmeli Y., Falagas M.E., Giske C.G., Harbarth S., Hindler J.F., Kahlmeter G., Olsson-Liljequist B. (2012). Multidrug-Resistant, Extensively Drug-Resistant and Pandrug-Resistant Bacteria: An International Expert Proposal for Interim Standard Definitions for Acquired Resistance. Clin. Microbiol. Infect..

[56] Borges N., Keller-Costa T., Sanches-Fernandes G.M.M., Louvado A., Gomes N.C.M., Costa R. (2021). Bacteriome Structure, Function, and Probiotics in Fish Larviculture: The Good, the Bad, and the Gaps. Annu. Rev. Anim. Biosci..

[57] Alvarez-Sieiro P., Montalbán-López M., Mu D., Kuipers O.P. (2016). Bacteriocins of Lactic Acid Bacteria: Extending the Family. Appl. Microbiol. Biotechnol..

[58] Imade E.E., Omonigho S.E., Babalola O.O., Enagbonma B.J. (2021). Lactic Acid Bacterial Bacteriocins and Their Bioactive Properties against Food-Associated Antibiotic-Resistant Bacteria. Ann. Microbiol..

[59] Rocchetti M.T., Russo P., Capozzi V., Drider D., Spano G., Fiocco D. (2021). Bioprospecting Antimicrobials from Lactiplantibacillus Plantarum: Key Factors Underlying Its Probiotic Action. Int. J. Mol. Sci..

[60] Pérez-Sánchez T., Balcázar J.L., Merrifield D.L., Carnevali O., Gioacchini G., de Blas I., Ruiz-Zarzuela I. (2011). Expression of Immune-Related Genes in Rainbow Trout (Oncorhynchus Mykiss) Induced by Probiotic Bacteria during Lactococcus Garvieae Infection. Fish Shellfish Immunol..

[61] Pérez-Sánchez T., Mora-Sánchez B., Vargas A., Balcázar J.L. (2020). Changes in Intestinal Microbiota and Disease Resistance Following Dietary Postbiotic Supplementation in Rainbow Trout (Oncorhynchus Mykiss). Microb. Pathog..

